# Characterization of Gelatin-Polycaprolactone Membranes by Electrospinning

**DOI:** 10.3390/biomimetics9020070

**Published:** 2024-01-25

**Authors:** Manuel Rodríguez-Martín, José Manuel Aguilar, Daniel Castro-Criado, Alberto Romero

**Affiliations:** Department of Chemical Engineering, Faculty of Chemistry, University of Seville, 41012 Seville, Spain; manrodmar7@gmail.com (M.R.-M.); jmaguilar@us.es (J.M.A.)

**Keywords:** tissue engineering, scaffolds, polycaprolactone, gelatin, nanofiber membranes, biomaterials

## Abstract

New advances in materials science and medicine have enabled the development of new and increasingly sophisticated biomaterials. One of the most widely used biopolymers is polycaprolactone (PCL) because it has properties suitable for biomedical applications, tissue engineering scaffolds, or drug delivery systems. However, PCL scaffolds do not have adequate bioactivity, and therefore, alternatives have been studied, such as mixing PCL with bioactive polymers such as gelatin, to promote cell growth. Thus, this work will deal with the fabrication of nanofiber membranes by means of the electrospinning technique using PCL-based solutions (12 wt.% and 20 wt.%) and PCL with gelatin (12 wt.% and 8 wt.%, respectively). Formic acid and acetic acid, as well as mixtures of both in different proportions, have been used to prepare the preliminary solutions, thus supporting the electrospinning process by controlling the viscosity of the solutions and, therefore, the size and uniformity of the fibers. The physical properties of the solutions and the morphological, mechanical, and thermal properties of the membranes were evaluated. Results demonstrate that it is possible to achieve the determined properties of the samples with an appropriate selection of polymer concentrations as well as solvents.

## 1. Introduction

Polycaprolactone, a linear synthetic biodegradable aliphatic polyester, has been widely used for the manufacturing of scaffolds. In contrast to other biomaterials used in scaffold development, it is relatively less expensive and can adopt different forms [1]. It is an FDA-approved polyester with excellent thermal stability, making it suitable for both load-bearing and non-load-bearing structures in tissue engineering applications [2]. As a result, it is susceptible to surface modifications, which can significantly change its properties like hydrophobicity and degradation. Since PCL is easy to process, it has been used to repair a variety of tissue defects [3]. However, hydrophobicity leads to non-optimal cell adhesion and proliferation. Therefore, PCL application can be carried out by combining or covering the pure PCL with other bioactive molecules [4]. On the other hand, gelatin, which is derived from collagen, the main protein in the extracellular matrix (ECM), which plays a crucial structural and mechanical role in skin, is a naturally occurring, biocompatible, and biodegradable substance. It is an important natural biopolymer in skin tissue engineering because it is recognized as an active agent that seems to keep informative signals like the RGD sequence, which promotes cell adhesion, differentiation, and proliferation [5,6]. However, collagen has poor mechanical strength. Making scaffolds for skin regeneration by mixing or coating mechanically stronger polymers, like PCL, with collagen would be a promising strategy. Some naturally occurring polymers can be electrospun into other structurally strong polymers to enhance their mechanical properties. The total hydrophilicity and cell proliferation rate of the nanofibers can be increased by mixing collagen with a synthetic hydrophobic polymer [7].

One of the main applications of PCL membranes is the development of scaffolds, which provide structural support for cell attachment and tissue development [8,9]. It must also carry out other functions like delivering, storing, and releasing active substances, as well as inducing specific cellular reactions that support the structural and mechanical integrity of the treated area. These must be stable and have the appropriate physical integrity to sustain sterilization as well as long-term storage [10]. Appropriate materials and effective fabrication methods are two major problems in tissue engineering. Different materials, including metals, ceramics, and polymers, have been studied to fabricate scaffolds over the past 20 years [11].

Polymeric scaffolds play an essential role in tissue engineering for cell adhesion, proliferation, and 3D new tissue formation and have great potential in a variety of tissues. Biocompatibility, biodegradability, and mechanical resistance are crucial scaffold parameters (i.e., the ideal pore size varies from 40 to 150 or 200 to 400 µm depending on the type of cells being sheltered) [12,13,14]. Biodegradable polymers are attractive candidates for scaffold materials because they degrade as new tissues are formed and can act as a material designed to repair or restore the functionalities of a defective biological system into a normal, healthy system [15]. Several techniques have also been investigated in this field, including a bottom-up approach using cell sheets [16], layer-by-layer cell assembly [17], and 3D printing [18]. The selection of the most cost-effective method that uses less time and energy while providing adequate architecture and stiffness is crucial. In this case, there are numerous reports on the fabrication of PCL-based scaffolds for different tissue engineering applications using electrospinning, rapid prototyping, phase separation, gas foaming, and fused deposition modeling [19,20,21]. The use of polymer nanofibers for tissue engineering relies on both their biochemistry properties and their ability to mimic the physical structure of native extracellular matrix at the nanoscale.

The process of electrospinning is a straightforward and adaptable method that is mostly used to create continuous micro- and nanofibers from polymer melts and solutions [22]. During the procedure, the charge accumulates, and it is driven to the surface of a developing polymeric droplet at the end of a metal needle when an electrical potential is applied between the polymer source and the collector. An electrically charged jet of solution-containing polymers erupts during electrospinning when the cohesive force of the solution, which is frequently dominated by surface tension, is overcome by the force of the electrical field. Electrostatic interactions between charges on adjacent segments of the jet force it to elongate as it approaches the collector plate. Meanwhile, the solvent evaporates, and the jet eventually solidifies into a fiber. Conventional electrospinning techniques yield extremely long fibers with diameters ranging from half to twice the average diameter along their length. These fibers could have a diameter that is far greater than the nanometer range [23]. Highly porous, flawless, and non-woven nanofibrous membranes can be produced with careful control over the parameters of the solutions and the operating environment. Thus, for a specific polymer to solubilize and undergo electrospinning transformation into nanofibers, solvent selection is essential. The solubility of the polymer in the solvent and the solvent’s boiling point, which indicates its volatility, are two crucial factors to take into account when choosing a solvent. Since volatile solvents have a lower boiling point and a faster rate of evaporation, they are usually the better option for dehydrating nanofibers as they move from the capillary tip to the collector surface. Very volatile solvents with low boiling temperatures, however, should be avoided since they may evaporate at the capillary tip and cause clogging and obstruction of the polymer solution’s flow rate. High-boiling-point solvents might not fully dehydrate before reaching the collector, which could lead to conglutination of nanofibers at boundaries or ribbon-like flat nanofiber morphologies [24,25]. Moreover, the solvents selected and their ratio affect the viscosity of the solutions. In this way, beads or beaded fibers are typically obtained when the viscosity is too low and the concentration of chain entanglements is also low. On the other hand, if the viscosity is too high, the constant spinning of fiber becomes difficult as a continuous polymer flow is inexistent [26]. Repulsive electrostatic forces promote the creation of fiber during the electrospinning process. The final architecture of the fibers is determined by jet instabilities caused by Coulomb interactions in the charged fluid jet. The polymer jet at the needle tip forms a Taylor cone because of these instabilities. This jet finally exists on the needle as a result of the forces acting on it, and thinning takes place [27].

The objective of this study was to study the influence of solvent ratios (acetic acid and formic acid) on the development and characterization of PCL and gelatin electrospun membranes. To achieve this, various solutions with different concentrations of PCL, gelatin, and solvents were prepared. The physical properties of solutions (viscosity, density, and conductivity) were evaluated in order to establish a relationship between these properties and the final results obtained. The physicochemical, microstructural, and mechanical properties of the elaborated membranes were also assessed. Processing conditions and proportions of solvents and polymer concentrations were selected after an exhaustive literature search, as shown in Table 1 and Table 2.

## 2. Materials and Methods

### 2.1. Materials

The materials used for the study were the synthetic polymer poly(ε-caprolactone) with a molecular weight of 80,000 g/mol and bovine gelatin protein type B, both provided by Sigma Aldrich Company (Saint Louis, MO, USA). On the other hand, 98% glacial acetic acid (AA) and 98% formic acid (FA), also provided by Sigma Aldrich, were used as solvents. These solvents were used since they are the most used for these polymers, in addition to being economical and having adequate volatility.

### 2.2. Membrane Manufacturing

#### 2.2.1. Preparation of Solutions

To carry out the study, seven solutions of 50 g each were prepared using acetic acid and formic acid as solvents in different proportions, with a PCL content of 12 wt.% in six of them and 20 wt.% in the other. In addition, gelatin type B at 8 wt.% was added to five of the solutions containing PCL at 12 wt.% (Table 3). These values were selected in accordance with an intermediate value for the literature study shown in Table 1 and Table 2.

Once the concentration of the polymers in each solution was determined, the corresponding amounts of solvents and solutes in each sample were weighed in glass containers in a fume hood due to the highly volatile nature of the solvents. To facilitate mixing, the solutions were kept on a magnetic stirrer for 12 h at 300 rpm [52]. At the end of the dilution, it was verified that the weight of the solute and solvent remained the same. If this was not the case, more solvent was added to replace the losses resulting from evaporation. Finally, the solutions obtained were stored in a refrigerator at 4 °C until the electrospinning process was carried out.

#### 2.2.2. Electrospinning Process

Bioinicia’s Fluidnatek LE-50 (Valencia, Spain) model electrospinning equipment was used for the preparation of PCL membranes, with or without gelatin [45]. Prior to the electrospinning process, the sample was removed from the refrigerator to temper it. Subsequently, ca. 6 mL of the polymer solution was loaded into a 10-mL syringe attached to a pump that exerted pressure such that the solution flowed at a fixed flow rate of 1 mL/h through a Teflon tube into a 22G-gauge (0.4 mm diameter) stainless steel needle, Nipro (Osaka, Japan). Between the needle and the collector used, separated by 15 cm, a potential difference of 15 kV was applied. The cathode (needle) was set at a voltage of 17 kV, while a voltage of −2 kV was applied to the anode (collector). The negative voltage application on the collector favors the projection and deposition of nanofibers on its surface. At the same time, to guarantee the homogeneity of the processing conditions, constant values of temperature and relative humidity were established, being 25 °C and 30%, respectively. Subsequently, the fibers will be deposited on a static cylindrical aluminum collector wrapped in aluminum foil to facilitate their extraction once the process is concluded, estimating a minimum processing time of two hours to appreciate the formation of a layer of nanofibers thick enough to be able to carry out the foreseen studies. Finally, the sheets will be removed from the collector and stored in closed bags in the refrigerator at 4 °C before proceeding with the characterization.

### 2.3. Solution Characterization

#### 2.3.1. Physical Properties

A digital densimeter, model Densito 30P (Mettler Toledo, Barcelona, Spain), was used to measure the density of the polymer solutions. Three measurements were taken at room temperature.

A digital conductometer model, the EC-Meter BASIC 30+ (Crison Instruments, Barcelona, Spain), was used to measure the conductivity of the samples. The measurement was performed by electrical equilibrium, and three measurements were taken at room temperature.

#### 2.3.2. Rheological Properties

The shear viscosity of the solutions was measured using an AR2000 rheometer (TA Instruments, New Castle, DE, USA). The measurements were carried out using a cone-plate sensor system made of steel with a diameter of 60 mm and an angle of 2°. The test was performed at room temperature, covering the sensor with a glass hood to prevent the escape of volatile gases. The selected program was steady-state flow, where the purpose was to obtain the viscosity curve (η) versus the shear rate ($\dot{\gamma }$) at which each point would be taken once equilibrium was reached. All curves were performed at shear rates ranging from 0.01 to 100 s^−1^. Since the evaluated solutions exhibited a Newtonian flow behavior, the mean value of the viscosity was taken.

### 2.4. Membranes Characterization

#### 2.4.1. Contact Angle Measurements

This test is useful to determine the wettability of the sample surface. The used equipment was a DSA25 drop-shape analyzer (Krüs, Hamburg, Germany). To carry out the measurements, a drop of a liquid, usually deionized water, with a volume of approximately 2 µL is placed on the sample surface for 10 s. During this period, images of the drop are taken to analyze the contact angle.

#### 2.4.2. Thermogravimetric Analysis (TGA)

For the thermal characterization of the structures obtained during electrospinning, samples of 12 wt.% PCL with acetic acid and formic acid in proportions of 1:1 (M1) and the same sample but with an addition of 8 wt.% gelatin (M4) were selected. The objective will therefore be to study how the presence of gelatin affects the thermal properties of the structures. In this sense, TGA measurements were carried out using a TGA Discovery (TA Instruments, New Castle, DE, USA) to study the thermomechanical stability of the membranes. In these analyses, 10 mg of the material were introduced into the equipment at 25 °C, and then the temperature was raised to 500 °C at a speed of 10 °C/min. During this test, the sample was kept in a nitrogen atmosphere.

#### 2.4.3. Scanning Electron Microscopy (SEM)

To carry out this test, Zeiss EVO equipment (Zeiss, Oberkochen, Germany) was used. Samples were observed at a 10 kV acceleration voltage and at a magnification between 500× and 2000×. A thin layer of gold was applied to the surface of the samples to give them conductive properties. The fiber size distribution was determined by considering 100 measurements of the fiber diameter using the digital tool FUJI ImageJ (Tokyo, Japan). At the same time, the porosity was measured using the same program, taking three measurements and calculating the average.

#### 2.4.4. Tensile Test

A dynamic-mechanical rheometer (DMA) model RSA3 (TA Instruments, New Castle, DE, USA) was used in this study, using a modification of the UNE-EN ISO 527-3:2019 standard [53]. First, five to six test specimens are previously prepared, cutting the membranes into pieces with dimensions of 60 mm long by 10 mm wide. Once the specimens have been cut, the polymeric membranes are carefully separated from the aluminum foil. The specimens are subjected to tensile stress at a speed of 0.083 mm/s, with 300 points per zone. Stress is applied until the specimens are broken. In this study, Young’s modulus (E), maximum tensile strength (σ_max_), and strain at break (ε_max_) of each electrospun membrane were evaluated.

#### 2.4.5. Statistical Analysis

At least three replicates of each measurement were carried out. Statistical analyses were performed using one-way analysis of variance (ANOVA, *p* < 0.05). The mean and standard deviation of each measurement were calculated.

## 3. Results and Discussion

### 3.1. Solution Characterization

The efficiency of the electrospinning process depends on the composition and physical properties of the solutions prepared. Therefore, these solutions were characterized by measuring viscosity, density, and conductivity. The results of these measurements are summarized in Table 4. It should be noted that it was not possible to measure the properties of sample M2 because of the high viscosity found due to the gelling process. In this sense, this sample is discarded for the electrospinning process.

Comparing M1 and M7 systems, an increase in viscosity with polymer concentration is observed due to an increase in the number of molecules and, consequently, their interactions and crosslinking [54]. In addition, considering the values observed for the M4 and M7 systems, PCL molecules provide higher viscosity than gelatin molecules due to their higher molecular weight. Likewise, it can be observed that viscosity also increases with the proportion of acetic acid present in the solution. The sample with the highest proportion of acetic acid (M3) shows a higher viscosity compared to other samples, even gelling in the case that the solvent was only acetic acid (M2). This may be an indication that it is possible to increase the viscosity without necessarily increasing the polymer concentration by modifying the solvents used. On the other hand, a slight increase in the density of the solutions is observed as the proportion of formic acid and the concentration of polymers increase. As for conductivity, some significant differences are observed. On the one hand, the systems with more components (M3, M4, and M5) have a higher conductivity than the others. This increase may correspond to a greater number of elements in the solution, which generate more dissolved ions and increase the charge intensity. In addition, an increase in conductivity is observed as the proportion of formic acid increases. This is consistent with the fact that the dielectric constant of formic acid (57.9) is higher than that of acetic acid (6.2). Between samples M1 and M7, which are the systems without gelatin, a slight decrease in conductivity is observed as the proportion of polymer increases as a consequence of the apolar character of this polymer. Finally, sample M6 shows the lowest conductivity compared to the other samples, possibly because it contains only one solvent while two solvents are used in the others. Therefore, it seems that the number of components directly influences the charges present in the solutions.

### 3.2. Membrane Characterization

#### 3.2.1. Contact Angle

**Figure 1:**
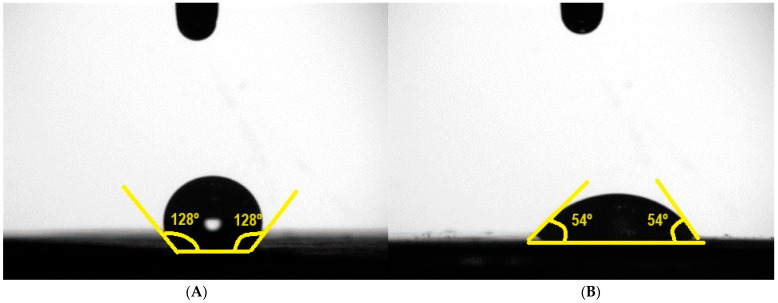
Results of the contact angle test of the M1 (**A**) and M4 (**B**) systems.

To evaluate how the presence of gelatin affects the hydrophobicity of PCL membranes, the contact angle values of three similar samples containing the same solvent ratio and the presence of gelatin (M1 and M4) were measured. Figure 1 shows the results of these tests.

M1 showed a contact angle value of 128° ± 6°, whereas M4 had a contact angle of 54° ± 20°. As previously mentioned, the presence of gelatin favors the wettability and bioactivity of PCL membranes. This can be experimentally reflected in the results obtained, where the presence of gelatin significantly reduces the surface contact angle, leading to a higher hydrophilic character, as can be observed in Figure 1. This leads to the fact that the incorporation of gelatin results in more hydrophilic membranes with adequate contact angles to improve cell adhesion [55,56]. In fact, for the case of muscle cells, it is estimated that cell enhancement is obtained for values between 40 and 60° [57].

#### 3.2.2. TGA

**Figure 2:**
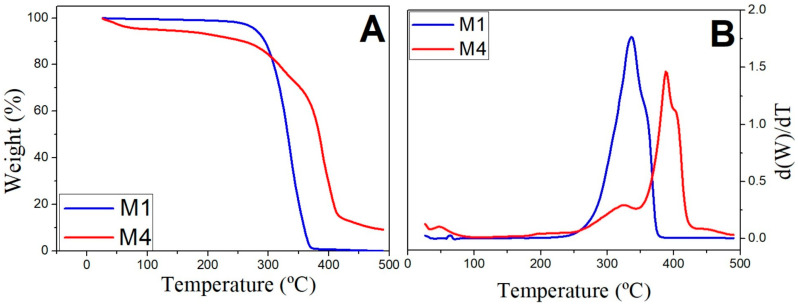
Thermogravimetric analysis test of the M1 and M4 systems (**A**) and their corresponding derivative signals (**B**) between 25 °C and 500 °C.

Figure 2A,B shows a thermogravimetric analysis of the M1 and M4 systems and their corresponding derivative signals, respectively.

As can be observed, the first thermal event occurs around 50–100 °C due to the evaporation of the volatile residues on the surface of the membranes. The second thermal event occurs between 250–400 °C for the PCL structure and around 250–350 °C for the PCL structure with gelatin, which may be due to the decomposition of the polymers, PCL in the case of M1, and the breaking of peptide bonds of gelatin in the case of M4 [28]. The coincidence of the peaks of M1 and M4 could indicate the formation of chemical bonds between gelatin and PCL [58]. In M4, a third peak of higher intensity shifted to the right (350–425 °C) is observed, which may be due to the decomposition of PCL, thus indicating that the presence of gelatin delays the thermal decomposition of PCL.

Comparing the results obtained by adding gelatin to the structures, a displacement to the right is obtained in the thermal events, improving the thermal stability of the scaffolds, possibly due to the formation of covalent bonds between the PCL and the gelatin [59].

#### 3.2.3. SEM

**Figure 3:**
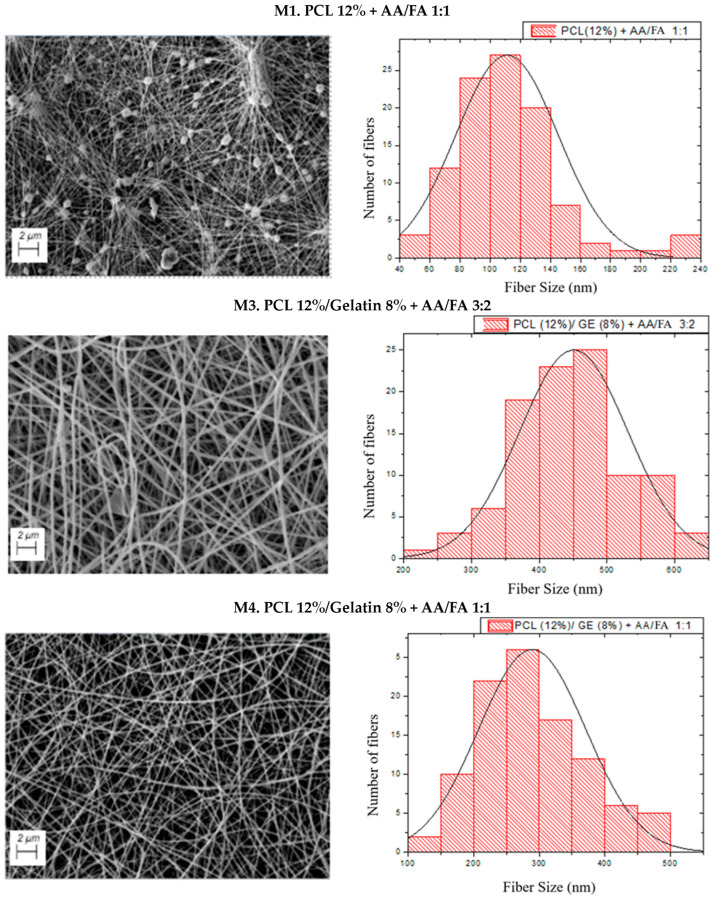

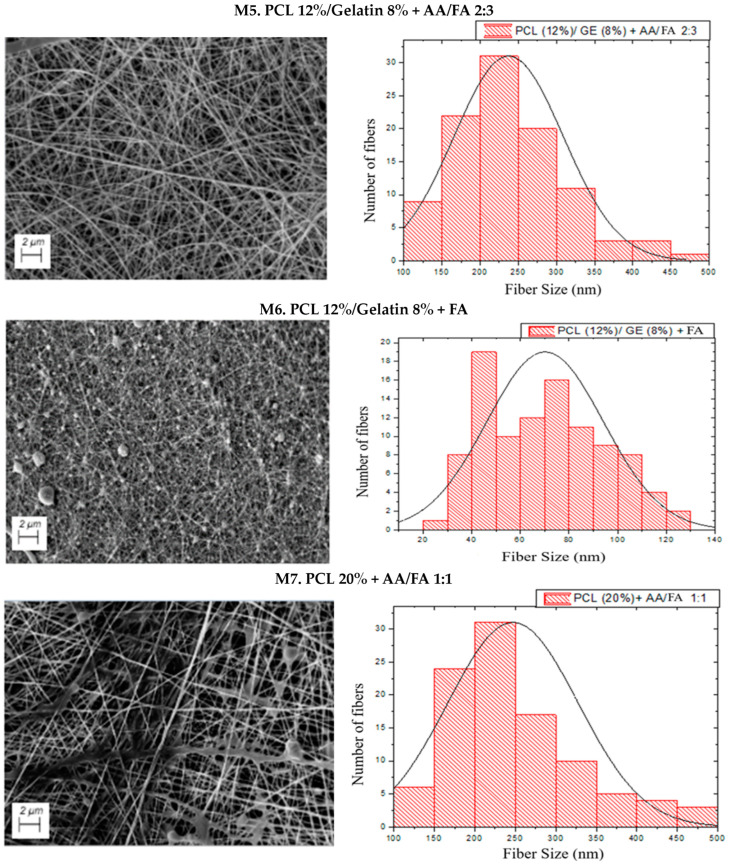
SEM images of each system and the fiber size distribution of each PCL structure made with different solvent ratios and polymer concentrations.

Figure 3 shows the SEM images and the fiber size distribution of each structure of PCL and PCL with gelatin using acetic acid and formic acid in different proportions.

SEM images show the formation of uniform and smooth fibers in M3, M4, and M5 structures, while in M1 and M6 samples, fine nanofibers with beads are observed. These defects may be due to the low viscosity of the solutions. On the other hand, in the 20 wt.% PCL sample (M7), homogeneous fibers with the presence of ribbons and few beads are observed, which may be due to a higher viscosity (0.682 Pa∙s) compared to its analogous with a lower PCL concentration (M1) (0.094 Pa∙s). Therefore, an increase in viscosity may generate wider and flatter fibers and, above all, reduce the number of beads. Furthermore, this may indicate that the optimum concentration to obtain homogeneous fibers lies between these two values. Hence, the fact that the systems produced, except for M3, have intermediate viscosity values between these limits is important. On the other hand, the systems that presented beads also showed low conductivity. This may be because low conductivity hinders the formation of the Taylor cone and, therefore, the electrospinning process. It could be verified that the formation of beads is conditioned not only by the viscosity but also by the conductivity of the system [60]. In contrast, the samples with higher conductivity showed more uniform and continuous fibers, possibly due to the formation of a more stable Taylor cone.

From the fiber size histograms, the average nanofiber diameters of each sample were obtained. These data are shown in Table 5, together with the porosity and the size of the beads.

Fiber diameter values were obtained for PCL membranes between 80 and 350 nm and from 50 to 500 nm for PCL with gelatin scaffolds. The fibers obtained in this study were thinner and showed a higher porosity than those obtained in the study carried out by Ren et al. (2017), in which the average diameters of the nanofibers were 200–600 nm [61], which means that cell migration occurs at higher speeds [62]. Therefore, it was possible to process fiber sizes suitable for their application in tissue engineering. An example of this can be found in the study of Lim et al. (2021), who designed scaffolds of aligned nanofibers in a satisfactory way for tissue engineering focused on the reconstruction of tendons and ligaments [46]. The average diameter of those electrospun PCL-gelatin-based nanofibers ranged from 200 to 800 nm, a similar range of values to the one obtained.

It can be observed that the solvent ratio significantly affects the diameter of the fibers. An increase in the proportion of acetic acid favors the formation of thicker but more homogeneous fibers. On the other hand, the presence of formic acid reduces the fiber diameter, although it can also favor the formation of defects. One of the main reasons is that the solvent ratio affects the viscosity of the solutions. In this way, beads or beaded fibers are typically obtained when the viscosity is too low and the concentration of chain entanglements is also low. On the other hand, if the viscosity is too high, the constant spinning of fiber becomes difficult as a continuous polymer flow is inexistent [26]. Moreover, the addition of gelatin significantly increased the porosity of the membranes, possibly due to the formation of more homogeneous fibers without defects that prevented the formation of agglomerated fibers.

**Figure 4:**
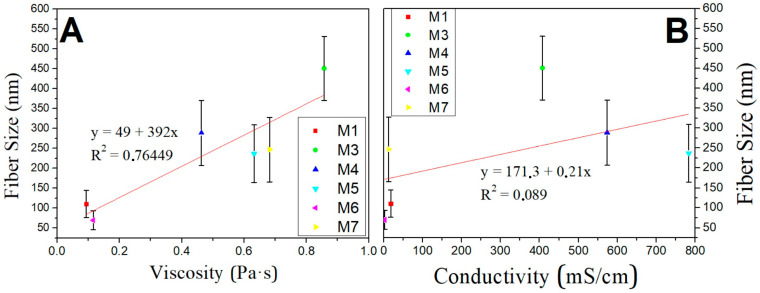
A graphic representation of fiber size and viscosity (**A**) and fiber size and conductivity (**B**) for the seven systems made.

To visualize the results obtained, Figure 4A,B graphically shows the relationship between fiber size and viscosity and fiber size and conductivity, respectively.

Figure 4A shows the aforementioned direct relationship between viscosity and the diameter of the fibers, together with the margin of error. It can be observed that there is a linear trend, so that with increasing viscosity, larger fiber sizes tend to be obtained, whereas there is no relationship between conductivity and fiber size.

#### 3.2.4. Tensile Tests

**Figure 5:**
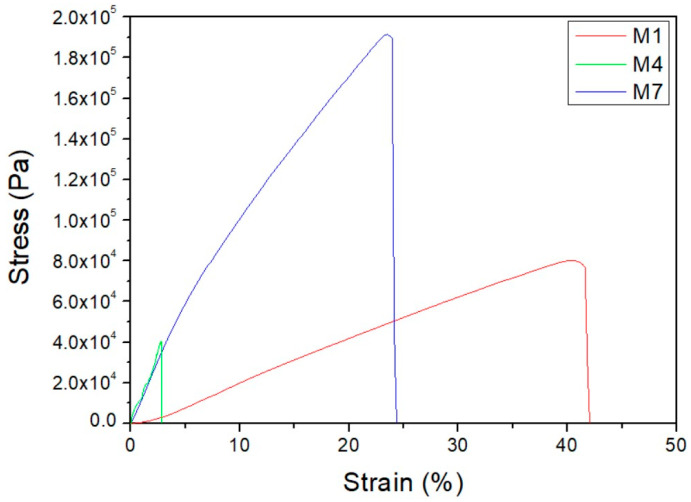
Stress–strain curves of the M1, M4, and M7 membranes.

This section intends to evaluate the mechanical properties of PCL and PCL with gelatin structures containing acetic acid and formic acid in different proportions. For these determinations, the tensile-to-rupture tests were carried out using die-cut sheets of known dimensions for each membrane. For this purpose, several sheets of each sample were prepared. However, it was not possible to separate the PCL with gelatin from the aluminum support in the M3, M5, and M6 samples because they were too thin, and their tests could not be carried out. Figure 5 shows the stress–strain curves of the membranes made, where it is observed that the M7 membrane shows a more resistant behavior while the curve of the M1 membrane presents a more ductile behavior. The M4 membrane could be separated from the support, although, like the M3, M5, and M6 membranes, it was very thin, which may be the reason for the very poor mechanical behavior shown in the graph.

For a better comparison, Table 6 shows Young’s modulus (E), maximum stress (σ_max_), and strain at break (ε_max_). From the results, it is observed that the 20 wt.% PCL membrane sample presents a higher maximum stress and a higher Young’s modulus. This may be because this structure had a higher proportion of homogeneous fibers with respect to the sample with a lower PCL concentration. Finally, it can be deduced from the results that a higher polymer concentration leads to stiffer but less deformable membranes. On the other hand, the incorporation of gelatin gives rise to more heterogeneous structures with high porosity and, consequently, worse mechanical properties, especially more fragile membranes.

Nevertheless, in this test, the thickness used was low, as the aim was to compare the mechanical properties of the specimens obtained in this study. In order to compare with other systems in the literature, it would be necessary to elaborate specimens of similar thickness.

## 4. Conclusions

The overall conclusion is that it is possible to successfully develop and characterize PCL and gelatin membranes using acetic acid and formic acid as solvents by electrospinning. In fact, the addition of gelatin to the PCL blends improved the surface properties, significantly reducing the contact angle and making the PCL films hydrophilic and with a contact angle suitable for muscle cell adhesion. Thermal characterization of the membranes verified that the addition of gelatin leads to a shift of the thermal events at higher temperatures to the right, which implies higher thermal stability.

Regarding morphological characterization, it can be concluded that both the viscosity and the conductivity of the precursor solutions play a fundamental role in the final microstructure of the membranes. In addition, the incorporation of gelatin increased both the fiber diameter and the porosity of the membranes, leading to membranes with worse mechanical properties.

Finally, it can be concluded from these results that the addition of gelatin to PCL membranes or scaffolds, with proper control of the solvents used, significantly improves their properties for their application in tissue engineering. Furthermore, in order to translate these results to their application in medicine, histological and functional evaluations are required.

## Figures and Tables

**Table 1:** Solvents used, polymer concentrations, and PCL electrospinning conditions.

Solvents	PCL Molecular Weight (kDa)	PCL Concentration (%)	Voltage (kV)	Needle-Collector Distance (cm)	Flow Rate (mL/h)	Needle Diameter (mm)	Reference
Acetic acid/Formic acid 1:1	85	15	25	15	1	0.5	[28]
Acetic acid/Formic acid 1:1	80	10	9	10	6	0.8	[29]
Acetic acid/Formic acid 1:1	80	15	7	10	6	0.8	[29]
Acetic acid/Formic acid 1:1	80	14	12; 16; 20	10; 15; 20	0.5; 1.25; 2	0.8	[30]
Acetic acid/Formic acid 1:1	80	18	20; 12; 16	15; 20; 16	0.5; 1.25; 2	0.8	[30]
Acetic acid/Formic acid 1:1	80	22	16; 20; 12	20; 10; 15	0.5; 1.25; 2	0.8	[30]
Acetic acid/Formic acid 1:1	80	26	20	25	10	0.8	[30]
Acetic acid/Formic acid 1:9	80	22	16–20	17.5	1.56	0.8	[31]
Acetic acid/Formic acid 3:7	80	12	15	17	0.2	-	[32]
Acetic acid/Formic acid/acetone 1:1:1	80	8; 10; 12	50	17	-	-	[33]
Acetic acid/Formic acid/acetone 1:1:1	45	14; 16; 18	50	17	-	-	[33]
Formic acid	80	14	17	13	0.5	0.82	[34]
Chloroform	80	12	24	19	1.56	0.8	[35]
Chloroform	80	14; 16; 18	8	15	0.5	-	[36]
Chloroform	80	10	13	-	6	1	[37]
Chloroform/dimethylformamide 7:3	80	17	9	20	1	0.8	[38]
Chloroform/ethanol 7:3	80	12	20	22	1	-	[32]
Chloroform/ethanol 8:2	80	8; 10;12	50	17	-	-	[33]
Chloroform/ethanol 8:2	45	14; 16; 18	50	17	-	-	[33]
Chloroform/ethanol/acetic acid 8:1:1	80	8; 10; 12	50	17	-	-	[33]
Chloroform/ethanol/acetic acid 8:1:1	45	14; 16; 18	50	17	-	-	[33]
Chloroform/methanol 12:1	80	12	16–20	17.5	1.56	0.8	[31]
Chloroform/methanol 24:1	80	12	16–20	17.5	1.56	0.8	[31]
Chloroform/methanol 6:1	80	12	16	19	1.62	0.6	[35]
Chloroform/methanol 6:1	80	8; 12	16–20	17.5	1.56	0.8	[31]
Chloroform/methanol 7,3:1	80	12	24	19	1.56	0.8	[35]
Dichloromethane	80	10	15	10	0.05; 0.1; 0.15; 0.2	0.4	[39]
Dichloromethane/methanol 4:1	80	16	12	20	5	0.5	[28]
Dimethylformamide/dichloromethane 7:3	55.6	10	15; 17; 20	15	0.5	0.3	[40]
Dimethylformamide/dichloromethane 1:1	80	15	17.5	15	1	0.9	[41]
Hexafluoroisopropanol	80	8	20	20	0.8	0.84	[42]
Hexafluoroisopropanol	80	14	14	22	1	0.8	[43]
Hexafluoroisopropanol	60	13	20	22	0.2	0.2	[44]
Hexafluoroisopropanol	45	16	14	14	0.4	0.5	[45]
Trifluoroethanol	-	6	5	20	0.05	-	[46]
Trifluoroethanol	80	10	20	15	1	-	[39]
Trifluoroethanol/dimethylformamide 3:1	80	10	27	-	1.5	0.8	[47]

**Table 2:** Solvents used, polymer concentrations, and PCL with gelatin electrospinning conditions.

Scheme	PCL Molecular Weight (kDa)	PCL Concentration (%)	Gelatin Concentration (%)	Gel Type	Voltage (kV)	Needle-Collector Distance (cm)	Flow Rate (mL/h)	Needle Diameter (mm)	Ref.
Acetic acid	80	8	40	A	15	15	0.2	-	[32]
Acetic acid	48–90	10	10	B	20	15	0.8	0.8	[48]
Acetic acid/formic acid 1:1	85	13.5; 12; 10.5; 9; 7.5; 6; 4.5; 3	1.5; 3; 4.5; 6; 7.5; 9; 10.5; 12	A	25	15	1	0.5	[28]
Acetic acid/formic acid 1:1	80	19.2	4.8	-	22	15	1.5	0.8	[30]
Acetic acid/formic acid 9:1	80	13.5; 12; 10.5; 9; 7.5; 6; 4.5; 3; 1.5	1.5; 3; 4.5; 6; 7.5; 9; 10.5; 12; 13.5	A	10	15	0.6	0.34	[49]
Formic acid	80	14	4; 2	A	25	13	1	0.82	[34]
Formic acid	70–80	10	2; 4; 6	-	18	10	0.5	-	[50]
Hexafluoroisopropanol	45	16	2; 4	B	14	14	0.4	0.5	[45]
Hexafluoroisopropanol	80	8	2; 4	A	15	15	0.8	0.84	[42]
Trifluoroethanol	-	6	10	-	5	20	0,05	-	[46]
Trifluoroethanol	80	10	10	A	10	13	2	-	[51]
Trifluoroethanol/acetic acid 1000:2	80	10	10	-	27	-	1.5	0.8	[47]

**Table 3:** Composition of each solution evaluated.

	Solvents	Molecular Weight PCL (kDa)	Mixture Concentration (%)	PCL Concentration (%)	Gelatin Concentration (%)
M1	AA/FA (1:1)	80	12	12	0
M2	AA	80	20	12	8
M3	AA/FA (3:2)	80	20	12	8
M4	AA/FA (1:1)	80	20	12	8
M5	AA/FA (2:3)	80	20	12	8
M6	FA	80	20	12	8
M7	AA/FA (1:1)	80	20	20	0

**Table 4:** Viscosity, density, and conductivity values of each solution obtained. Different letters mean significant differences (*p* < 0.05).

	Systems	Viscosity (Pa·s)	Density (g/cm^3^)	Conductivity (μS/cm)
M1	PCL 12% + AA/FA (1:1)	0.094 ± 0.001 ^a^	1.123 ± 0.001 ^A^	18.17 ± 0.65 ^β^
M2	PCL 12%/GE 8%+ AA	-	-	-
M3	PCL 12%/GE 8% + AA/FA (3:2)	0.857 ± 0.022 ^e^	1.145 ± 0.006 ^C^	407.67 ± 3.51 ^γ^
M4	PCL 12%/GE 8% + AA/FA (1:1)	0.463 ± 0.002 ^c^	1.143 ± 0.001 ^C^	573.67 ± 2.08 ^δ^
M5	PCL 12%/GE 8% + AA/FA (2:3)	0.632 ± 0.005 ^d^	1.158 ± 0.001 ^D^	783.33 ± 5.51 ^ε^
M6	PCL 12%/GE 8% + FA	0.116 ± 0.004 ^b^	1.212 ± 0.001 ^E^	2.84 ± 0.03 ^α^
M7	PCL 20% + AC/FA (1:1)	0.682 ± 0.002 ^d^	1.131 ± 0.001 ^B^	12.81 ± 1.30 ^β^

**Table 5:** Summary of the morphological properties of each membrane evaluated. Different letters mean significant differences (*p* < 0.05).

	Systems	Fiber Size (nm)	Porosity (%)	Size of Beads (μm)
M1	PCL 12% + AA/FA (1:1)	111 ± 34 ^b^	47.0 ± 0.8 ^c^	1.05 ± 0.38
M2	PCL 12%/GE 8% + AA	-	-	-
M3	PCL 12%/GE 8% + AA/FA (3:2)	451 ± 80 ^d^	36.2 ± 2.3 ^b^	-
M4	PCL 12%/GE 8% + AA/FA (1:1)	289 ± 82 ^c^	56.2 ± 2.3 ^d^	-
M5	PCL 12%/GE 8% + AA/FA (2:3)	237 ± 72 ^c^	33.9 ± 34 ^b^	-
M6	PCL 12%/GE 8% + FA	70 ± 24 ^a^	44.2 ± 1.6 ^c^	0.56 ± 0.39
M7	PCL 20% + AA/FA (1:1)	247 ± 81^c^	27.7 ± 1.1 ^a^	-

**Table 6:** Summary of the mechanical properties parameters of PCL and PCL with gelatin membranes. Different letters mean significant differences (*p* < 0.05).

	Systems	ε_max_ (%)	σ_max_ (kPa)	E (kPa)
M1	PCL 12% + AA/FA (1:1)	38.0 ± 3.3	82.5 ± 3.3	22.6 ± 1.8
M4	PCL 12%/GE 8% + AA/FA (1:1)	5.5 ± 0.2	40.6 ± 12.0	66.6 ± 7.8
M7	PCL 20% + AC/FA (1:1)	21.5 ± 2.0	181.0 ± 9.1	89.6 ± 0.65

## Data Availability

Data are contained within the article.
